# Theoretical Design of the Scattering-Based Sensor for Analysis of the Vehicle Tailpipe Emission

**DOI:** 10.3390/mi11121085

**Published:** 2020-12-07

**Authors:** Sama Molaie, Paolo Lino

**Affiliations:** Polytechnic University of Bari, 70125 Bari, Italy; paolo.lino@poliba.it

**Keywords:** emission exhaust, optical particle counter, tailpipe, particulate mass, particulate number

## Abstract

Measurement regulations demand, among other requirements, the reduction of particulate matter emissions from diesel engines. Considering this, the establishment of a new measurement instrument for periodic emission control and detection of the Diesel Particulate Filter (DPF) performance after the vehicle exhaust is necessary. To fulfil these requirements, this paper proposes the design of a new, simple, low-weight layout after the vehicle tailpipe. In order to check the operation condition of the proposed sensor, different factors such as the temperature (−10 to 50 ℃) humidity (60%), and flow rate of the sampled emission (laminar condition) are considered. The proposed layout uses an optical particle counter as a portable instrument for real-time detection of the particle concentration after exhaust of the internal combustion engine.

## 1. Introduction

The combustion process of a diesel engine can be defined as unsteady turbulent diffusion combustion [1] and it operates with a non-homogeneous air–fuel mixture at a high temperature inside the combustion chamber [2]. Diesel engines are more effective in terms of fuel consumption if compared to their counterpart spark ignition (SI) engines. However, the high emission particulate matter (PM) produced by diesel engines is a primary challenge that needs to be tackled by scientists and researchers [3].

The first directive for the regular roadworthiness check of exhaust gas emission testing dates back to 1992 [4], and its main objective was to limit a vehicle’s emissions to within specific values throughout its life. Considering the difficulties in the evaluation of type-approval procedures for testing gaseous emissions and fumes emitted by all types of vehicles, the availability of simple and cheap testing equipment and methods would be useful. Additionally, the PM emissions of diesel engines are much higher than gasoline engines [5], which has pushed legislators to introduce more stringent particulate emission regulations.

For this reason, the fulfilment of the European Commission requirements necessitates the adoption of the best available technology for Diesel Particulate Filters (DPFs) [6], together with regular checking of the emissions to monitor the current performance of the DPFs. Designing low-weight and cheap equipment to detect emissions after the tailpipe would enable a fast and affordable analysis of the DPF performance and faults. Currently, the periodic emission check for diesel vehicles is based on the smoke opacity, which has a low correlation with particulate mass or particulate number emissions. Furthermore, available opacimeters have an accuracy which is not suitable for measuring low smoke emission levels [7].

One of the standard instruments for detecting the number concentration is the condensation particle counter (CPC), which uses light scattering to count the particles after magnifying their size [8]. The number concentration can easily be determined by measuring the pulses from each particle and the sampling flow rate. Since selecting an appropriate saturation ratio is essential, the saturator and condenser temperature remains in a limited range. Therefore, it is always a challenge to use the device in an outdoor temperature [9].

The TSI Nanoparticle Emission Tester (NPET) Model 3795 [10] is designed to detect the number concentration of the combustion engine (e.g., a diesel engine). The sensor is based on a compatible sampling nozzle with tailpipes, a dilution system, a catalytic stripper for removing the volatile particles, and a CPC for counting the PM. Additionally, the system fulfills the requirement of the Swiss regulation SR941.242. The SENSORS company introduced another condensation particle number (CPN) [11], which follows the European Real Driving Emissions (RDE) Particle Number (PN) Portable Measurement Systems (PEMS) measurement requirements. The main concerns regarding both systems are their bulky dimensions, high weight, and cost. For these reasons, the condensation particle counter is not appropriate for applications located far from the laboratory environment. Diffusion Chargers (DCs) [12] are mainly used as compact, robust, and lightweight sensors for particle number concentration measurement. Generally, DCs consist of particle charging and subsequent detection of the current [8]. The accuracy of the method can be estimated to be about 25% [8] to 30% [13].

Recently, the first prototype for adapting diffusion chargers for detection of the number concentration of a raw engine exhaust was successfully designed [14]. However, it was important to clean the sensor and provide continuous maintenance because of direct exposure of the sensor to PM emissions, the high temperature of exhaust emissions, and variation of the flow rate. An alternative version using an integral ejector pump was proposed [15]. The sensor provides a fixed flow rate and protects the corona from exposure to pollution. Rüggeberg et al. [16] introduced a modified version of the sensor. The system can operate at more than 150 °C and does not require dilution. However, the sensor has a high weight (about 10 kg) and a large size. Recently, M. A. Schriefl et al. [17] presented the experimental characteristics of two pulsed-mode diffusion charging sensors (Naneos Partector and a custom-built pulsed-mode modular diffusion charger (PMDC)) for detection of the particle number. The result of the tailpipe measurement confirmed the capacity of PMDC for periodical test measurements of vehicles.

However, there are limitations to the successful use of DC sensors. Firstly, the sensor response considerably depends on the size of the particle. In this case, different fuel blends and engine loads affect the system accuracy. Secondly, the sensor cannot meet high precision counting requirements [18]. Thirdly, the system is not able to directly detect the number concentration. Therefore, additional information on the particle size distribution for converting the measured current into the particle number concentration is required. This process needs signal manipulation, and until now, there has been no calibration standard to ensure the accuracy of detection [18]. In this framework, DC sensors are no longer a candidate for detection of the DPF performance and particle concentration of the vehicle exhaust.

This paper proposes the theoretical design of a new cheap and simple detection system for measuring the PM concentration after the tailpipe. However, even though the accuracy and sensitivity of the sensing device are essential features, they must be assessed considering the whole detection system, also including the exhaust sampling subsystem. In fact, since the quality of the sample transportation and dilution can affect the particulate matter structure and cause transformations, it can be considered a more critical aspect of the detection process [19].

Considering the proper design of the sampling system after the exhaust, selecting a real-time, low-cost mobile sensing system with an acceptable accuracy is another crucial factor. There are a variety of suitable methods for detection of the PM mass concentration. The methods of detection of the PM engine exhaust can be divided into two main categories, i.e., direct detection and indirect detection. Gravimetric methods [20] and microbalance methods [21] fall in the first category. Gravimetric approaches [8] are time-consuming, labor intensive, and do not provide temporal information. Moreover, these large devices are usually stationary and do not provide a real-time measurement [8]. Microbalance methods are not useful for the detection of vehicle emissions due to problems with humidity, temperature, and overloading. Moreover, the device is expensive and its dimensions are quite large.

Another category of methods relies on indirect detection of the particulate matter mass concentration [22]. The scanning mobility particle sizer (SMPS), aerodynamic particle scanner (APS), and optical particle counters (OPCs) are based on an indirect detection method. By assuming that the density of the particles is known, these instruments automatically calculate the mass concentration of the particulate matter using measurements of the particulate number concentration within different sizes [23,24]. Despite the accurate detection of the mass concentration, the main limitations of APS and SMPS are their high cost and huge size.

Demands for using optical particle counters due to the possibility of detection in real-time by using a compact, simple, and inexpensive instrument are increasing. OPC sensors represent a widely adopted low-cost solution for detecting the PM mass and number concentration in both indoor and outdoor environments [21,25]. Generally, for scattering-based methods, an accuracy of 30% is assumed [8]. Therefore, the accuracy of the proposed sensor is much lower than that of professional instruments employed for mass concentration detection. However, the final selection of the sensor is decided based on monitoring purposes and the available budget.

The available systems for the periodic detection of particular matter and determination of DPF are quite expensive. For example, CPCs and DC sensors (testo DiSCmini, West Chester, PA, USA) [26] have costs of (without a sampling system) 10 and 15 k$, respectively. The proposed sensor is relatively cheap, with a components cost of about 1200$ (with a dilution system), and it is able to determine the DPF faults and performance. The main aim of this research is to design a cheap, low-weight, and compact system based on scattering techniques to determine the mass concentration of the exhaust emission directly after a diesel vehicle. The proposed sensor should ensure the reliable detection of high emitters (i.e., DPF faults) with a limited degree of complexity.

This paper is organized as follows. Section 2 describes the measurement principle of the optical particle counter, while Section 3 focuses on the sampling process. Section 4 shows how to calculate the total temperature of the diluted sampling system. The results of the humidity and vapor partial pressure detection are described in Section 5. Section 6 presents the calibration method for the proposed sensor. Finally, Section 7 draws some conclusions.

## 2. Principle of the Optical Particle Counter

### 2.1. Structure of the Optical Particle Counter

A constant-flow pump enables the sampled particulate matter to flow through the optical sensor chamber and finally exit the outlet pipe. Inside the sensing chamber, an inlet nozzle directs the flow through the accumulated laser beam. The round inlet sampling pipe of the OPC is designed in a way that the laminar flow through the sampling pipe is guaranteed.

Generally, inside the measurement chamber, the laser beam is accumulated by different lenses or mirrors. A variety of lasers with different wavelengths can be used in the measurement chamber to accommodate more particle sizes.

### 2.2. Observation Volume of Sensing Cells in OPC

The volume of observation can be identified at the intersection of the laser light beam and the sampling pipe and is characterized by a cylindrical shape. The sensing volume orthogonally placed with respect to the photodetector and the presence of a single particle in the observation volume are common assumptions that are applied for optical particle counters [27]. Since the final aim of the equipment is to detect the real concentration of the particulate matter, it should be assumed that one particle passes through the observation volume at a time. If $w_{l}$ is the laser beam diameter and d is the diameter of the inlet nozzle, the observation volume or volume of the sensing cell $V_{\mathrm{obs}}$ can be calculated as follows:

(1) $$V_{\mathrm{obs}}=\;\pi \;\frac{d^{2}}{4}\;w_{l}.$$

### 2.3. Probability of Coincidence

Optical particle counters are widely used in research and industry to detect the particle mass and number concentration. Considering the size detection of the particle, one of the critical errors of the OPC is the coincidence of particles, which is the simultaneous presence of more than one particle inside the observation volume [28,29]. Raasch and Umhauer (1984) [30] theoretically explored the effect of coincidence error in the detection process of optical particle counters.

In this theory, particles are assumed as point masses with no preferred position and no interaction with each other while they pass through the observation volume. Whitby and Willeke (1979) [31] expressed this error and suggested two solutions for decreasing coincidence errors:

1. Decreasing the observation volume [27],
2. Diluting the sampling particle flow to the OPC.

Both of these methods are analyzed in this paper, and the best solution is selected for using the OPC after the exhaust.

### 2.4. OPC Device for Detection of the Particulate Matter

There is a continuously increasing interest in the mobile and low-cost monitoring of PM [32]. OPC devices are mainly used to detect the indoor and outdoor air quality, and some research groups are testing them for different aerosol sources in various environments [33,34]. Three main factors, i.e., the temperature, humidity, and inlet PM flow concentration, should be considered for a proper selection of technical specifications of the OPC for each environment [35].

The OPCs available on the market operate on the same physical principle, but differ in terms of construction, design, and cost [36,37]. Finally, the detection of the PM size distribution requires knowledge of the relationship between the intensity of the scattered light and the size of the particles, for which a calibration step must be carried out [37].

### 2.5. Diesel Particulate Matter Concentration

Diesel engines display a higher level of diffusion than gasoline engines because of their cost, reliability, and high performance. Despite these advantages, they are considered to be a major source of pollutants in the urban environment [5]. In particular, the production of PM of diesel engines is about ten times greater than that of gasoline engines, representing a severe threat to human health and the environment. This is the main reason why much recent research related to the detection of pollutant emissions has been conducted.

Usually, the normal-logarithmic size distribution [38] of the diesel particulate matter has three modal structures: Nucleation mode; accumulation mode; and coarse mode. The peak value of the particulate matter number distribution is contained in the nucleation mode, while the maximum value of the particulate matter mass distribution is found in the accumulation mode. A small fraction of the particulate matter remains in the third coarse mode, which is not generated during the combustion process [38].

### 2.6. Number Concentration and DPF

**Figure 1:**
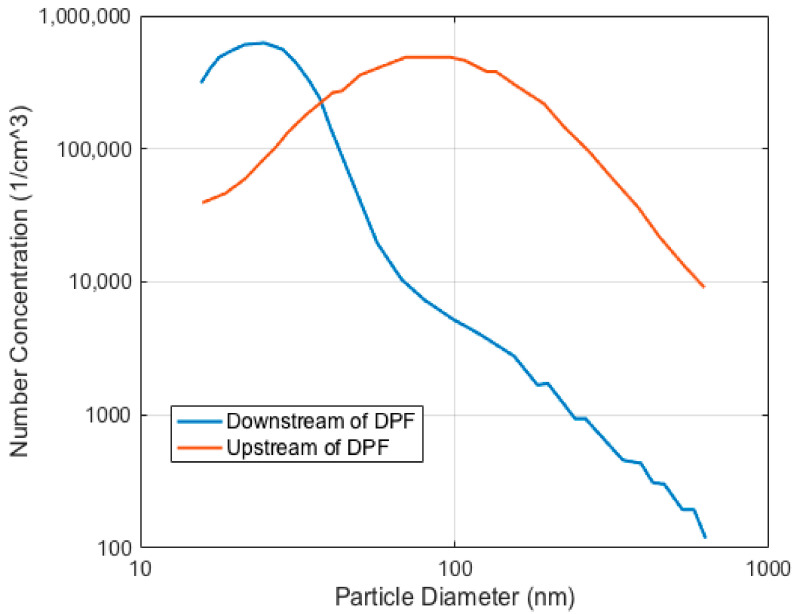
Typical size distribution of the particulate matter downstream and upstream of the Diesel Particulate Filter (DPF).

The U.S. Environmental Protection Agency (EPA) established restrictive regulations to limit the PM emissions of diesel engines. To fulfil the prescriptions, many researchers have aimed to develop new technologies for after-treatment systems. DPFs were introduced during the early years of the century to remove PM and control emissions [5], guaranteeing a reduction [39] of two orders of magnitude of PM in the accumulation mode of particulate matter (see Figure 1).

The reduction of the number and mass concentration of the PM after the diesel exhaust thanks to the use of new technology is obviously desirable. Considering the limitations of optical sensors in the detection of particulate matter [8], the concentration of particulate matter after the tailpipe is still high in relation to coincidence error. The use of OPC sensors after the tailpipe requires a reduction of the observation volume (to be able to detect one particle at a time), or, as an alternative, a decrease of the PM concentration after the exhaust (which can be achieved by diluting the raw exhaust emission with air).

### 2.7. OPC-N3 Alphasense Company

A wide variety of low-cost optical particle sensors from different companies (e.g., Dylos (Riverside, CA, USA), AlphaSense (Essex, UK), and TSI (Shoreview, MN, USA)) are available on the market. One sensor for detection of the particulate mass and number after the exhaust is the OPC-N3 sensor produced by AlphaSense Company, which is commonly used for air quality monitoring and is characterized by a good compromise between cost and accuracy. Table 1 summarizes the main characteristics of OPC-N3.

It is worth noting that, since the minimum detection size of OPC-N3 is 0.35 μm, the focus of the proposed research will mostly be on the accumulation mode rather than the nucleation mode. The accumulation mode contains solid carbonaceous particles resulting from incomplete fuel combustion. The size and number of the accumulated particles depend on the combustion process, fuel oxidation, the condensation of species, etc. [40]. Additionally, OPC-N3 cannot wholly detect the total mass distribution of particulate matter after the vehicle tailpipe, so further developments should be considered in future research. Therefore, this research aims to design a low-weight, low-cost, and accurate system for detection of the PM after the exhaust of the internal combustion engine, by taking into account the influence of different operating conditions, such as the temperature and humidity. As the particle size distribution of different vehicles may change slightly, calibration tests should be performed for different vehicles. There are several exhaust emission particles in the nucleation mode, which OPC cannot detect. However, this is not a concern, as we focus on the particulate matter mass concentration (accommodation mode) to determine the DPF performance.

Optical particle counters are used to detect the concentration of airborne particles in ground- based or balloon-based atmospheric studies. Non-spherical soot particles containing individual spherules are always found in the atmosphere [41]. These soot aggerates are created by the combustion process (e.g., diesel engines). Non-spherical particles are usually defined by the equivalent diameter, which is the diameter of the spherical particle, defined as the equal size measurement of the particle under consideration. Assuming that dust particles are spheres, laboratory measurements were in good agreement with theoretical modeling of the scattering properties of non-spherical particles by the use of Mie theory [42]. The Grimm aerosol sensor provides an excellent capability for detecting the particulate mass concentration by using the light scattering method [43]. The sensor is compact, durable, and only requires minimal maintenance. The accuracy of the OPC-N3 sensor compared to the Grimm sensor under a constant environmental condition is about 11% to 14% over the range of the PM1.0 mass concentration. The sensor accuracy also slightly increases (16% to 24%) with a rise of the PM2.5 mass concentration [44]. Generally, for both values, the concentration of the PM at 20 ℃ and 40% relative humidity is greatly underestimated [44] in comparison with the sensor.

However, for using the sensor after the tailpipe, the sampling emission of the raw exhaust can affect the accuracy of the optical system due to the high temperature, high humidity, and deposition of particles on optical components. An appropriate dilution system prevents the condensation of water vapor on the wall of the sampling pipe, exhaust tailpipe, and optical sensing unit, thus improving the accuracy detection of the system [8]. Moreover, dilution can decrease the temperature and concentration of the sampling emission to an acceptable range for the optical sensor, in order to avoid hazardous effects on the sensor performance and reliability. In this framework, a simplified sampling system using ejectors can dilute the raw exhaust and provide a cost-effective measurement.

### 2.8. Detection of Particulate Matter after the Exhaust Using OPC

The concentration of the particulate matter emission from the tailpipe, even if using DPF, is still too high for accurate detection by an optical particle sensor. Accurate detection of the size distribution requires a low coincidence error, which can be obtained by reducing the volume of observation.

**Figure 2:**
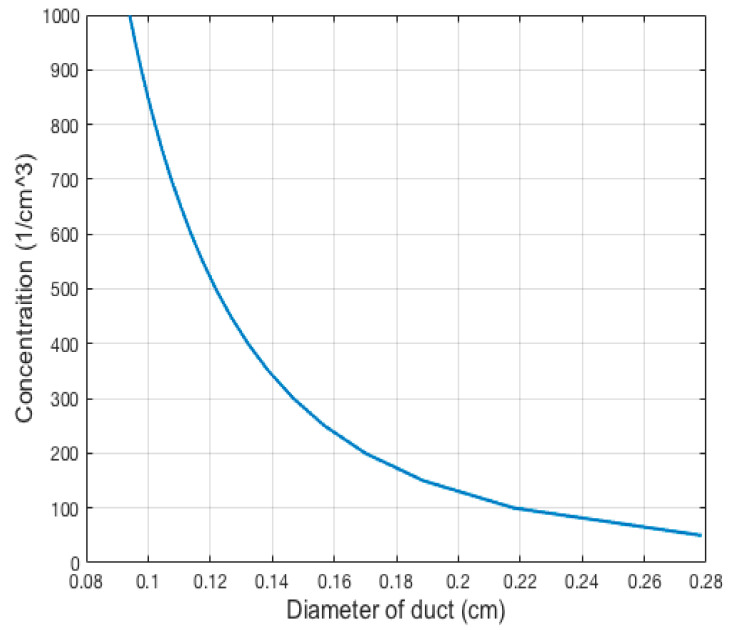
Number concentration vs. the inlet duct diameter.

Considering that the maximum real number concentration of a diesel engine for a particle of 0.35 μm is about 500 $\left(1/{\mathrm{cm}}^{3}\right)$ [39], it is possible to estimate the dimension of the volume of observation required to obtain the flow of one particle at a time. Figure 2 shows the relationship between the concentration and diameter of the inlet sampling emission to obtain one particle in the volume of observation. For the defined cylindrical volume of observation, it is assumed that the diameter of the laser $w_{l}$ is 0.5 mm bigger than the inlet duct diameter. In fact, the maximum measurable concentration is limited by the minimum diameter of the observation volume that can be realized. Considering the maximum value of concentration of about 500 $\left(1/{\mathrm{cm}}^{3}\right),$ shown by Figure 2, the needed reduction can be achieved with an inlet duct diameter of 0.121 cm.

To obtain the proposed observation volume, the diameter of the laser beam should be reduced to the value of 0.171 cm, which is simply achieved by using different lenses. The main problem is the difficulty in building an inlet duct satisfying the requirements. The solution consists of diluting particulate matter after the exhaust with purge air.

Table 1 shows the maximum coincidence probabilities of 0.84 and 0.28, and maximum particulate number concentrations of 1000 $\left(1/{\mathrm{cm}}^{3}\right)$ and 0.500 $\left(1/{\mathrm{cm}}^{3}\right),$ respectively. For a number concentration of 500 $\left(1/{\mathrm{cm}}^{3}\right)$, the probability of coincidence is still too high, while determination of the exact size of particulate matter is one concern of the detection system. Therefore, the best solution for reducing the coincidence error is to decrease the number concentration of particulate matter to the feasible value of around 60 $\left(1/{\mathrm{cm}}^{3}\right)$, which can be obtained with a dilution factor of 8 using Equation (2). The real and diluted concentration can be expressed using the following formula:

(2) $$C_{d}=\frac{C_{r}}{DF},$$

where $C_{d}$ is the concentration of the diluted emission, $C_{r}$ is the real concentration of the exhaust emission, and DF is the dilution factor.

In addition to decreasing the number concentration, it is necessary to monitor the temperature, pressure, and humidity variations of the diluted particulate matter emission before the optical particle counter, which should be in an acceptable range to fulfill the optical sensor operating requirements.

## 3. Sampling Process

The sampling process, as an essential part of the sensing system, can provide the accuracy and validation of the detection system. The condition of the diluted exhaust emission in the inlet probe of the measurement chamber, in terms of the temperature and humidity, is a primary issue for the detection process of OPC. The accurate detection of particulate matter requires an inlet nozzle pressure and temperature within the range of 1 bar and −10 to 50 ℃, respectively [35]. The exhaust gas flow rate of the inlet duct is another major issue. To avoid the effect of humidity and vapor condensation on the performance of the optical particle counter, the total relative humidity of the sampling diluted gas should be less than 75% [45].

In addition, the deposition of volatile organic compounds (VOC) on particles can affect the PM measurement. However, the target of the study is not to develop a laboratory-grade device, but rather a robust, inexpensive, and portable system for a rough estimation of the PM concentration. In this framework, any system that would also consider the evaporation of VOC to stabilize PM measurement would then violate the requirements to be compact, portable, and inexpensive.

### 3.1. Ejector and Dilution Factor

Ejector dilutors are extensively used in exhaust measurement because of their simple design [46,47]. Ejector dilutors work by mixing a limited amount of raw sample emission with a fixed amount of dilution gas or air. In the proposed design, to decrease the concentration of the sampled emission flow before the optical sensor, the particulate matter of exhaust emissions undergoes rapid dilution with ambient air after exiting the tailpipe.

**Figure 3:**
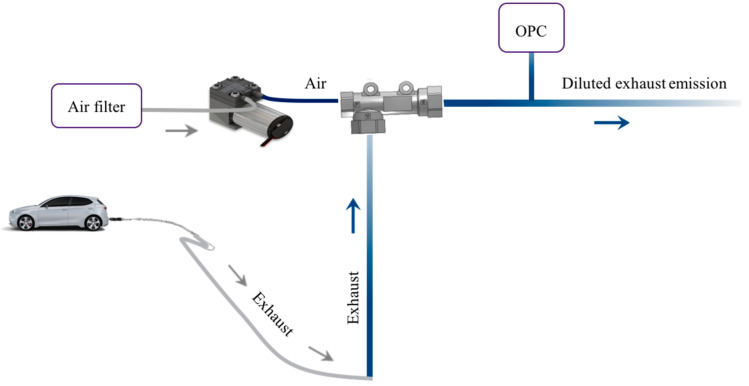
Layout of the system using one ejector for the detection of particulate matter.

The suction part of the ejector is connected to the steel tube which is inserted into the tailpipe, while the fluid part of the ejector is connected to the air filter to remove any additional particulate matter entering the detection system. The use of a single head pump before the fluid part of the ejector is required to obtain a constant flow rate of particulate matter in the mixing zone. Figure 3 shows the overall layout of the detection system using the optical sensor after the vehicle tailpipe.

The ejector is placed between the optical sensor and the tailpipe of the vehicle to mix the raw exhaust with environmental air.

### 3.2. Parameters of the Mix Condition

**Figure 4:**
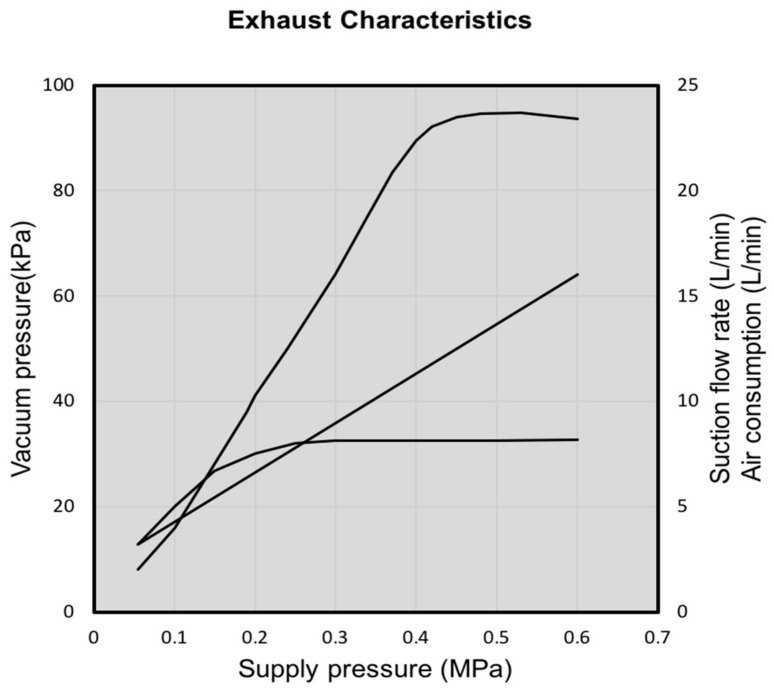
Characteristic curve of the ejector.

The calculation of the total flow rate in the mix zone of the ejector for determination of the dilution factor requires knowledge on the volumetric flow rate in the suction part of the ejector (exhaust gas) and supply part of the ejector (purge air). Both of them can be defined by using the characteristic curve and selecting the operation pressure of the supply part of the ejector. Among the cheap ejectors available on the market, the ZH05DSA-06-06-06 ejector with a small throat section of 0.5 mm was selected. Figure 4 shows the characteristic curve of the selected ejector. Different sample compositions, temperatures, and pressures imply the use of ejector dilutors in different locations along the sampling line. Table 2 shows the specifications of the compact and light weight ZH05DSA ejector produced by SMC company [48].

The calculations of the total volumetric flow rate of the mix condition and dilution factor are shown in Table 3. The total flow rate is calculated by the sum of the exhaust and air flow rate with the following formula:

(3) $$Q_{T}\;\cdot \rho_{T}=\;Q_{\mathrm{exhaust}\;}\cdot \;\rho_{\mathrm{ex}}+Q_{\mathrm{air}\;}\cdot \rho_{\mathrm{air}}$$

where $Q_{T}$ is the total volumetric flow rate of diluted particulate matter sampling in a mix condition, $Q_{\mathrm{exhaust}}$ is the volumetric flow rate of the raw exhaust in the suction part of the ejector, and $Q_{\mathrm{air}}$ is the volumetric flow rate of the environmental air in the supply part of the ejector. Here,$\;\rho_{T}$, $\rho_{\mathrm{ex}}$, and $\rho_{\mathrm{air}}$ are the total density of the diluted particulate matter, density of the exhaust emission, and density of the air, respectively. It should be mentioned that to simplify the calculation, the total density and exhaust density are assumed to be equal to the air density.

The dilution factor in Table 3 is defined as the ratio of the air flow rate and exhaust flow rate, and is calculated by the following formula:

(4) $$DF=\frac{Q\mathrm{air}}{Q\mathrm{exhaust}},$$

By looking at Table 3, to achieve the maximum value of the dilution factor, it is reasonable to choose a 6 bar relative pressure for the supply part of the ejector. In addition, after setting the dilution factor and choosing the supply pressure of the ejector, the pump operating point is established based on the given pressure and flow rate. To make ambient air flow inside the ejector, the pump should be placed before the supply part of the ejector.

**Figure 5:**
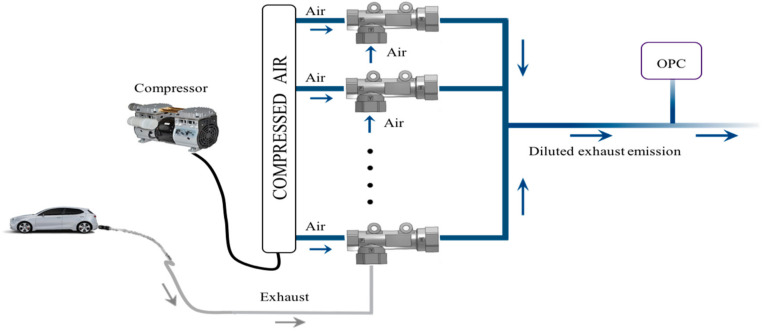
Layout of the system after the exhaust using parallel ejectors.

Additionally, by using the proposed layout, the concentration of the exhaust sampled emission decreases, but it is not enough to obtain accurate detection by OPC. Considering the OPC-N3 technical specification (Table 1) and maximum detectable concentration of particulate matter (0.500 $1/{\mathrm{cm}}^{3}$) at a particle size of 0.35 μm, the coincidence probability is still high for the presence of each particle in the observation volume. In this framework, to increase the dilution factor up to 8, a different layout including more ejectors in parallel for detection of the particulate matter after the tailpipe is proposed (Figure 5).

All ejectors except the last one use ambient air in the supply and suction part. The fraction of the raw exhaust emission is sucked from the tailpipe by the suction part of the last ejector. To dilute the exhaust emission with ambient air, a proper operating point is set for the compressor. As previously mentioned, it is chosen based on the selected supply pressure of the ejector and volumetric flow rate. However, it is necessary to use two air filters before the compressor and in the air suction part of the ejectors.

In particular, to obtain a dilution factor equal to 8, a supply pressure of 0.5 MPa is selected, based on the characteristic curve of the ejector. The dilution factor can be calculated by the following formula, while the ambient air flows in all the ejectors, except for the suction part of the last ejector:

(5) $$DF=\frac{\sum_{i=1}^{N}Q_{\mathrm{supply}\_\mathrm{air}}+\sum_{i=1}^{N-1}Q_{\mathrm{suction}\_\mathrm{air}}}{Q_{\mathrm{exhaust}\;\mathrm{gas}}},$$

where $Q_{\mathrm{supply}\_\mathrm{air}}$ is the volumetric flow rate of the supply air, $Q_{\mathrm{suction}\_\mathrm{air}}$ is the volumetric flow rate of the air in the suction part of the ejector, and $Q_{\mathrm{exhaust}\;\mathrm{gas}}$ is the volumetric flow rate of the emission gas in the suction part of the last ejector. The calculation of $Q_{\mathrm{Total}-\mathrm{Air}}$ is made as follows:

(6) $$Q_{\mathrm{Total}-\mathrm{Air}}\cdot \rho_{\mathrm{air}}=\left(n-1\right)\cdot Q_{\mathrm{Total}-\mathrm{ejector}}\cdot \rho_{\mathrm{air}}+\;Q_{\mathrm{Supply}-\mathrm{ejector}}\cdot \rho_{\mathrm{air}},$$

where $Q_{\mathrm{Total}-\mathrm{Air}}$ is the total volumetric flow rate of air, n is the number of ejectors in parallel, and $Q_{\mathrm{Supply}-\mathrm{ejector}}$ is the volumetric flow rate of the supply part of the last ejector. It should be mentioned that, in each step, $Q_{\mathrm{Total}-\mathrm{ejector}}$ for each ejector is the sum of $Q_{\mathrm{suction}}$ and $Q_{\mathrm{supply}}\;$.Table 4 shows the total volumetric flow rate, dilution factor, and operating point of the compressor using the ZH05DSA-06-06-06 ejector. In the proposed layout, to obtain a dilution factor equal to 8, four ejectors in parallel are connected.$\;Q_{\mathrm{air}\_\mathrm{compressor}}$ presents the final volumetric flow rate of the selected compressor.

**Figure 6:**
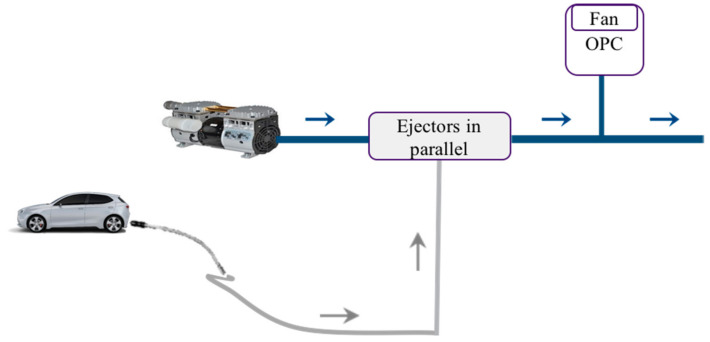
Final layout of the optical particle counter (OPC) perpendicular to the main flow rate.

The final value of the volumetric flow rate after four ejectors is about 69.66 (L/min). Connecting the optical particle sensor to the main tube enables the sampling of a proportion of the exhaust emission to be used in the OPC. The indirect use of airflow allows the OPC fan to regulate the flow rate at the appropriate value suitable for the optical sensor (Figure 6).

Table 5 shows the total cost of components for designing the proposed sensor. The sensing part of the system is quite cheap (about 340$). However, to increase the accuracy of the system and durability, the dilution of the raw exhaust emission with environmental air was proposed.

In the proposed sensor, different factors of the mixed emission (such as the humidity, temperature, etc.) can be theoretically determined. However, using the additional sensors for the detection of these parameters is not recommended due to this increasing the cost and complexity of the system.

## 4. Temperature of the Inlet Mixture Emission of the Optical Sensor

As highlighted previously, one of the most important factors to be considered is the temperature of the mixture emission after the ejectors and before the optical sensor. Standard working conditions of the optical sensor require a temperature of the sampling emission of the ejectors discharge between −10 and 50 ℃, as shown in Table 1. The mass flow rate in the mixing point is constant for all temperature and humidity conditions, and can be calculated by the following formula:

(7) $$\;\;\;G_{\mathrm{mix}}=G_{\mathrm{suction}}+G_{\mathrm{supply}},$$

where $G_{\mathrm{mix}}$ is the mass flow rate after mixing, $G_{\mathrm{suction}}$ is the mass flow rate at exhaust suction, and $G_{\mathrm{supply}}$ is the mass flow rate of dilution air. The temperature $T_{\mathrm{mix}}$ $\left(℃\right)$ of the gas mixture and downstream of the ejector can be calculated considering the adiabatic mixing and mass balance equation to describe the condition before the optical sensor:

(8) $$T_{\mathrm{mix}\;}G_{\mathrm{mix}}=T_{\mathrm{air}-\sup}G_{\mathrm{air}-\sup}+\left(T_{\mathrm{air}-\mathrm{suc}}G_{\mathrm{air}-\mathrm{suc}}+\;T_{\mathrm{exhaust}-\mathrm{suc}}G_{\mathrm{exhaust}-\mathrm{suc}}\right),$$

where $T_{\mathrm{air}-\sup}$ is the air temperature in the supply part of the ejector; $G_{\mathrm{air}-\sup}$ is the overall supply air mass flow rate of the four ejectors; $T_{\mathrm{air}-\mathrm{suc}}$ is the air temperature in the suction part of the ejector, which is equal to $T_{\mathrm{air}-\sup}$; and $G_{\mathrm{air}-\mathrm{suc}}$ is the air mass flow rate in the suction part of the $\left(n-1\right)$ ejectors. Moreover, $T_{\mathrm{exhaust}-\mathrm{suc}}$ is the temperature of the exhaust emission flowing from the engine to the suction part of the last ejector, and $G_{\mathrm{exhaust}-\mathrm{suc}}$ is the mass flow rate of the exhaust emission of the engine in the suction part of the last ejector. At each step, the mass flow rate G can be computed as follows:

(9) G=Q·ρ,

where Q is the volumetric flow rate. As an approximation, the total mixture gas and emission sampling density are considered equivalent to the density of air.

**Figure 7:**
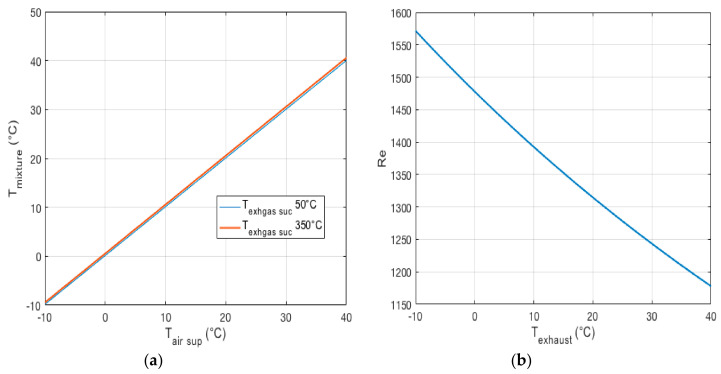
(**a**) Temperature in a mix condition vs. ambient temperature. (**b**) Reynolds number vs. temperature.

To calculate the temperature before the optical sensor in different operating conditions, the ambient air temperature was varied in the range of −10 to 40 ℃, while the temperature of the engine exhaust gas ranged between 50 and 350 ℃. Figure 7a shows the temperature of the gas mixture for different operating conditions.

From Figure 7a, it is clear that any change in environmental temperature can directly affect the temperature of the gas mixture after the ejector. However, the temperature after the ejector is less than 50 ℃, and an extra cooler before the optical counter is not necessary.

### Effect of the Temperature on the Laminar Flow of the Inlet Sampling

Using the small pump or fan of the OPC, the mixture of the emission exhaust gas and air flows into the inlet sampling probe, and then enters the observation volume of the optical sensor. In the observation volume, the scattered light intensity is detected by the photodetector, and the sampled mixture then exits the outlet of the OPC. Accurate measurement of the particulate matter concentration requires a laminar flow of the inlet sampling of the OPC, ensured by a proper Reynolds number value. The Reynolds number must always be less than 2300 to ensure a laminar flow [49] of the mixture sampling.

The total volumetric flow rate of the mixture sampling can be calculated by using the optical sensor technical specifications in Table 1. The flow velocity of the inlet mixture sampling can be calculated using the volumetric flow rate and the inlet diameter of the OPC (6 mm). Finally, the Reynolds number can be calculated given the density ρ of the mixture flow in the discharge of the ejectors (pressure of 1 bar and temperature between −10 and 40 ℃) and the dynamic viscosity $\vartheta \left(T\right)$ of the mixture sampling flow (only depending on the temperature).

Figure 7b shows the dependency of the Reynolds number on the temperature of the mixture flow, which varies between −10 and 40 ℃. It is worth noting that the Reynolds number is always below 2300, which confirms that the condition of the inlet sampling probe flow is always laminar.

## 5. Humidity for the Optical Sensor

Humid air can affect the behavior of the optical particle counter based on the light scattering principle in different ways. One possibility is the change of its performance due to the dependency of the particle refractive index on the relative humidity [45].

Secondly, humidity can affect electrical components by creating resistive bridges between each of them [45]. Thirdly, hygroscopic growth of the particles (like sodium chloride) with the relative humidity increase causes a significant large positive artefact in particle mass measurement [50]. In addition, if the humidity is about 100%, it is likely to detect liquid droplets as particles, downgrading the accuracy of the detection system. To avoid the formation of liquid droplets in the sampling probe before the optical sensor, the partial vapor pressure of the diluted sampling emission after ejectors should always be lower than the saturation vapor pressure, at the mixture temperature. The saturation vapor pressure is defined as the pressure of the vapor when it is in thermodynamic equilibrium with the liquid phase, for a specific temperature. The saturation pressure of the vapor mainly depends on the temperature.

**Figure 8:**
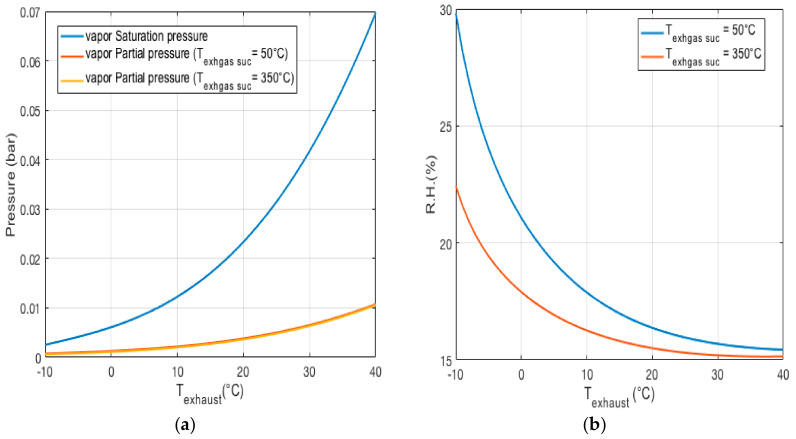
(**a**) Vapor partial pressure and vapor saturation pressure vs. temperature of the discharge ejector. (**b**) Humidity vs. temperature in the mix condition.

The particle mass and number concentration can be overestimated while the relative humidity is in the range of 70–75% [45], or even 60% [51]. Furthermore, it should be mentioned that the relative humidity inside the measurement chamber is not the same as that in the sampling probe before the optical sensor (Figure 6). The reason for this is the increase of temperature inside the chamber due to the electronics circuits when the OPC is functioning [52]. The first calculation of the vapor partial pressure was conducted by considering the temperature of the exhaust emission in the range of 50 to 350 ℃ (Figure 8a). To avoid any condensation, the partial vapor pressure of the sampling inlet emission must always be lower than the saturation pressure of the vapor. For the next step, the humidity of the sampled emission at different exhaust temperatures was calculated, and the results are shown in Figure 8b.

### Calculation of the Partial Pressure and Relative Humidity

For the calculation of the vapor partial pressure and relative humidity after the ejectors, the temperature, pressure, and relative humidity of the environmental air in the supply inlet are considered to be in the range of −10 ℃ to 40 ℃, 5 bar, and 50%, respectively. However, the variation of the relative humidity in the environmental air cannot affect the results.

Given the volumetric flow rate of the supply part of the ejector and the density, it is possible to calculate the supply mass flow rate. The mass of the vapor in the sampled air can be determined by considering the humidity of the air, and through the calculation of the wet air mixing ratio x.

Finally, the total mass of the $H_{2}O$ in the system $M_{H_{2}O-\mathrm{tot}}$ can be calculated as follows:

(10) $$M_{H_{2}O-\mathrm{tot}}=M_{H_{2}O-\mathrm{air}-\mathrm{supply}}+M_{H_{2}O-\mathrm{air}-\mathrm{suction}}+M_{H_{2}O-\mathrm{gas}-\mathrm{suction}},$$

where $M_{H_{2}O-\mathrm{air}-\mathrm{supply}}$ is the mass flow rate of water vapor in the air supply part of the ejectors, $M_{H_{2}O-\mathrm{air}-\mathrm{suction}}$ is the mass flow rate of the $H_{2}O$ in the air suction part of the ejectors, $\mathrm{and}\;M_{H_{2}O-\mathrm{gas}-\mathrm{suction}}$ is the mass flow rate of the $H_{2}O$ in the sampled exhaust emission that passes through the suction part of the ejector.

To compute the total mass flow rate of the dry air $M_{dry-tot}$, the following formula can be used:

(11) $$M_{\mathrm{dry}-\mathrm{tot}}=M_{\mathrm{air}-\mathrm{dry}-\mathrm{supply}}+M_{\mathrm{air}-\mathrm{dry}-\mathrm{suction}}+M_{\mathrm{gas}-\mathrm{dry}-\mathrm{suction}},$$

where $M_{air-dry-supply}$ is the mass flow rate of the dry air in the supply part of the ejectors, $M_{air-dry-suction}$ is the mass flow rate of the dry air in the suction part of the ejectors, and $M_{gas-dry-suction}$ is the mass flow rate of the dry emission of exhaust in the suction part of the last ejector.

Since the total mass flow rate of the dry air and water vapor is known, it is possible to calculate the water mixing ratio of the air–exhaust gas mixture, after ejectors and before the optical sensor, as follows:

(12) $$x_{\mathrm{mix}}=\frac{M_{H_{2}O-\mathrm{tot}}}{M_{\mathrm{dry}-\mathrm{tot}}}.$$

To calculate the relative humidity and partial vapor pressure after the ejectors, the values of 350 and 50 ℃ were considered for the exhaust emission. Figure 8a shows the saturation vapor pressure and partial pressure of the vapor while the temperature of the emission mixture changes between −10 and 40 ℃.

From Figure 8a, it can be observed that there is no water droplet in the observation volume, while the partial vapor pressure is always below the saturation pressure. The partial pressure of the vapor in the mix condition after ejector $P_{H2O-\mathrm{mix}\;}$ can be calculated as

(13) $$P_{H2O-\mathrm{mix}\;}=P_{\mathrm{STP}}\;\cdot \;10^{-5}\;\cdot \;\frac{x_{\mathrm{mix}}}{\left(x_{\mathrm{mix}}+0.622\right)},$$

where $P_{\mathrm{STP}}$ is the standard ambient pressure equal to 101,325 Pa. The relative humidity RH can be calculated by

(14) $$RH=\left(\;\frac{P_{H2O-\mathrm{mix}\;}}{P_{\mathrm{SAT}}}\right)\cdot \;100,$$

where $P_{\mathrm{SAT}}$ is the saturation pressure of the vapor only depending on the gas mixture temperature. Figure 8b shows the relationship between the humidity of the diluted exhaust emission in the mix condition after ejectors and before the optical sensor, for different temperatures of ejector discharge.

## 6. Calibration Process

One prerequisite for using a light scattering-based sensor for an evaluation of the DPF performance is the availability of a traceable and appropriate calibration method. In order to obtain an accurate measurement, it is necessary to determine the relationship between the scattered light intensity distribution and particle size distribution. The planned testing setup consists of a particle source, a reference sensor, and the designed PM sensor. In particular, a Standard Combustion Aerosol Generator (SCAG) is planned to be used as an exhaust source. It produces real combustion particles with a selected size distribution, which are stable and reproducible [53]. This solution is preferable as a soot particle source in the calibration process if compared to diesel engines, which require large spaces and involve high management costs. As a reference instrument for calibration, a scanning mobility particle sizer can be applied. Moreover, further examination of the mass concentration can be assessed by the standard gravimetric method or a micro soot sensor.

## 7. Conclusions

Among the sources of pollutants, diesel engines are recognized as one of the major sources of PM pollution of the last decades. Verification of the PM emission during a vehicle’s life and the identification of any defect of the after-treatment system, such as DPF, can be achieved through the regular measurement of tailpipe emissions.

There exists a wide spectrum of expensive instruments for testing the tailpipe emission, e.g., CPC in stationary test benches. These professional instruments are very accurate, but due to their high price and bulky structures, are not suitable for regular checking of the PM emission after the vehicle tailpipe. There are some drawbacks of the use of DC sensors for monitoring of the DPF performance. The system detection response is mainly influenced by differences in the particle size. DC sensors also lack appropriate calibration. Their system has a high weight and a scale size that is not small [16].

To address the increasing demand for the real-time detection of PM after the tailpipe, the theoretical design of a compact, low-weight, and affordable system including an OPC device after the tailpipe of the vehicle has been proposed. Due to the limitation of particle size detection of the OPC, the accumulated mode of diesel particulate matter can be considered in the proposed research. In this framework, we mostly focus on the mass concentration, rather than the number concentration. However, the accuracy limitation can be improved through proper calibration and by optimizing the data process [54].

To increase the accuracy of the optical detection system, it is necessary to reduce the coincidence error. For this purpose, one of the solutions is to dilute the exhaust emission without any physical changing of the OPC structure. In the present work, the dilution factor of 8 is chosen to decrease the maximum concentration of the exhaust emission to the value of 60 ($1/{\mathrm{cm}}^{3}$), which decreases the coincidence probability and increases the accuracy of the detection system. Considering this, a layout including four ejectors in parallel and one compressor to dilute the emission exhaust gas of the engine tailpipe has been designed. In order to use an OPC after the proposed layout, different parameters, such as the partial vapor pressure, humidity, and temperature of the inlet sampling emission before the optical sensor, were calculated.

The proposed solution based on an OPC and ejectors seems to be a cheap, small, and low-weight alternative to current systems employing for regular emission checking and detection of the DPF performance. In addition, the detection system is quite simple and can monitor the mass concentration in real-time. It should be mention that practical measurements should be scheduled for better evaluating the system performance under real operation conditions of the vehicle engine. The proposed system also requires proper calibration using accurate devices to detect the mass concentration of the particulate matter. In future research, advancements are expected in terms of ensuring a practical detection size by appropriate calibration and by changing the OPC structures.

## Figures and Tables

**Table 1:** OPC-N3 technical specifications [35].

Technical Data	OPC-N3
Number of bins	24
Size range $\left(\mu m\right)$	0.35 to 40
Total flow rate (L/min)	5.5
Temperature range $\left(℃\right)$	−10 to 50
Humidity range $\left(\%\mathrm{rh}\right)$	0–95
Max coincidence probability (concentration at 10^6^ (particles/lit))	0.84
Max coincidence probability (concentration at 500(particles/lit))	0.24
Weight $\left(g\right)$	<105

**Table 2:** Ejector specifications.

Technical Data	ZH05DSA-06-06-06
Fluid	Air
Operating temperature range $\left(℃\right)$	−5 to 50
Operating pressure range $\left(\mathrm{MPA}\right)$	0.1 to 0.6
Nozzle nominal size $\left(\mathrm{mm}\right)$	0.5
Maximum suction flow rate (L/min)	8.26
Air consumption (L/min)	16
Weight $\left(g\right)$	5

**Table 3:** Calculation of the flow rate and dilution factor.

Parameter	Values					
Pressure supply $\left(\mathrm{PM}\right)$	0.1	0.2	0.3	0.4	0.5	0.6
Flow rate of the exhaust (L/min)	5	7.5	8.15	8.15	8.15	8.26
Flow rate of air (L/min)	4.25	6.60	8.95	11.30	13.65	16
Dilution factor	0.85	0.88	1.09	1.38	1.67	1.93
Total flow rate after the exhaust (L/min)	9.25	14.1	17.10	19.45	21.80	24.26

**Table 4:** Dilution factor and flow rate of the second layout.

Parameters	Values
Number of ejectors	4
$Q_{\mathrm{Total}-\mathrm{Air}}\;\left(L/\min\right)$	69.66
$Q_{\mathrm{exhaust}}\;\left(L/\min\right)$	8.15
DF	8.54
$Q_{\mathrm{air}-\mathrm{compressor}}\;(L/\min)$	45.21

**Table 5:** The component cost of the system.

Optical Layout	List of Components	Numbers	Price [$]
Sampling system	Ejectors and tubes	4	76.6
	Pneumatic Double Y tube	2	29.42
Dilution system	Compressor	1	382.40
	Pressure switch	1	51.95
	Pneumatic filter	2	218
	One touch manifold	1	8.68
Optical sensing system	OPC-N3	1	340
Conjunction system	Accessories	-	90.15
	Total cost		≈1200

## Nomenclature

**Symbols for general parameters**	**Definitions**
$V_{\mathrm{obs}}$	Observation volume
$w_{l}$	Laser beam diameter
d	Diameter of the inlet nozzle
$C_{d}$	Concentration of the diluted emission
$C_{r}$	Real concentration of the exhaust emission
df	Dilution factor
n	Number of ejectors in parallel
$G_{\mathrm{mix}}$	Mass flow rate after mixing
$G_{\mathrm{suction}}$	Mass flow rate at exhaust suction
$G_{\mathrm{supply}}$	Mass flow rate of dilution air
G	Mass flow rate
Q	Volumetric flow rate
**Symbols for parameters of the fist layout**	**Definitions**
$Q_{T}$	Total volumetric flow rate of diluted sampling
$Q_{\mathrm{exhaust}}$	Volumetric flow rate of the raw exhaust
$Q_{\mathrm{air}}$	Volumetric flow rate of the environmental air
$\rho_{T}$	Total density of the diluted particulate matter
$\rho_{\mathrm{ex}}$	Density of the exhaust emission
$\rho_{\mathrm{air}}$	Density of the air
**Symbols for parameters of the second layout**	**Definitions**
$Q_{\mathrm{supply}\_\mathrm{air}}$	Volumetric flow rate of the supply air
$Q_{\mathrm{suction}\_\mathrm{air}}$	Volumetric flow rate of the suction air
$Q_{\mathrm{exhaust}\;\mathrm{gas}}$	Volumetric flow rate of the emission gas
$Q_{\mathrm{Total}-\mathrm{Air}}$	Total volumetric flow rate of air
$Q_{\mathrm{Supply}-\mathrm{ejector}}$	Volumetric flow rate of the supply (last ejector)
$Q_{\mathrm{Total}-\mathrm{ejector}}$	Volumetric flow rate of the supply + suction
$Q_{\mathrm{air}-\mathrm{compressor}}$	Volumetric flow rate of the compressor
**Symbols for calculation of the temperature**	**Definitions**
$T_{\mathrm{mix}}$	Temperature of the gas mixture
$T_{\mathrm{air}-\sup}$	Air temperature in the supply ejector
$T_{\mathrm{air}-\mathrm{suc}}$	Air temperature in the suction ejector
$T_{\mathrm{exhaust}-\mathrm{suc}}$	Exhaust temperature suction emission
$G_{\mathrm{air}-\sup}$	Overall supply air mass flow rate of ejectors
$G_{\mathrm{air}-\mathrm{suc}}$	Air mass flow rate of the suction (n-1) ejectors
$G_{\mathrm{exhaust}-\mathrm{suc}}$	Exhaust mass flow rate of the suction ejector
**Symbols for calculation of the humidity**	**Definitions**
$M_{H_{2}O-\mathrm{tot}}$	Total mass of the $H_{2}O$ in the system
$M_{H_{2}O-\mathrm{air}-\mathrm{supply}}$	Mass flow rate of water vapor in air supply ejectors
$M_{H_{2}O-\mathrm{air}-\mathrm{suction}}$	Mass flow rate of the $H_{2}O$ in air suction ejectors
$M_{H_{2}O-\mathrm{gas}-\mathrm{suction}}$	Mass flow rate of $H_{2}O$ in the emission suction ejector
$M_{\mathrm{dry}-\mathrm{tot}}$	Total mass flow rate of the dry air
$M_{\mathrm{air}-\mathrm{dry}-\mathrm{supply}}$	Mass flow rate of the dry air in supply ejectors
$M_{\mathrm{air}-\mathrm{dry}-\mathrm{suction}}$	Mass flow rate of the dry air in suction ejectors
$M_{\mathrm{gas}-\mathrm{dry}-\mathrm{suction}}$	Mass flow rate of the dry emission in the suction ejector
$x_{\mathrm{mix}}$	Water mixing ratio of the air–exhaust gas mixture
$P_{H2O-\mathrm{mix}\;}$	Partial pressure of the vapor in the mix condition
$P_{\mathrm{STP}}$	Standard ambient pressure
RH	Relative humidity
$P_{\mathrm{SAT}}$	Saturation pressure of the vapor
